# Commonly used estimates of the genetic contribution to disease are subject to the same fallacies as bad luck estimates

**DOI:** 10.1007/s10654-019-00573-8

**Published:** 2019-10-22

**Authors:** Jonas Björk, Tomas Andersson, Anders Ahlbom

**Affiliations:** 1 Division of Occupational and Environmental Medicine, Lund University, Box 117, 221 00 Lund, Sweden; 2 Clinical Studies Sweden, Forum South, Skåne University Hospital, Lund, Sweden; 3 Unit of Epidemiology, Institute of Environmental Medicine, Karolinska Institutet, Stockholm, Sweden; 4 Center for Occupational and Environmental Medicine, Stockholm County Council, Stockholm, Sweden

**Keywords:** Epidemiology, Genetic studies, Public health, Etiologic fraction

## Abstract

The scientific debate following the initial formulation of the “bad luck” hypothesis in cancer development highlighted how measures based on analysis of variance are inappropriately used for risk communication. The notion of “explained” variance is not only used to quantify randomness, but also to quantify genetic and environmental contribution to disease in heritability coefficients. In this paper, we demonstrate why such quantifications are generally as problematic as bad luck estimates. We stress the differences in calculation and interpretation between the heritability coefficient and the population attributable fraction, the estimated fraction of all disease events that would not occur if an intervention could successfully prevent the excess genetic risk. We recommend using the population attributable fraction when communicating results regarding the genetic contribution to disease, as this measure is both more relevant from a public health perspective and easier to understand.

**Electronic supplementary material:**

The online version of this article (10.1007/s10654-019-00573-8) contains supplementary material, which is available to authorized users.

## Introduction

In 2015, the “bad luck” hypothesis in cancer development was put forward, which initially claimed that a majority of the variation in cancer risk is due to randomness [1]. The origin of the hypothesis was the observed strong correlation (*R*^2^ = 0.66 = 66% explained variance) across cancer types between the total number of stem cell cellular divisions, assumed to represent the randomness, and lifetime cancer risk. The subsequent scientific debate was intense, and highlighted how measures based on analysis of variance are inappropriately used for risk communication [2, 3]. The notion of “explained” variance is not only used to quantify randomness, but also to quantify genetic and environmental contribution to disease in heritability coefficients (*h*^2^) [4]. Examples can be found both in scientific writing, e.g. “heritable factors were estimated to account for 42% of prostate cancer risk” [5], and in media, e.g. “About a third of all cancer cases can be blamed on inherited genes, a giant study finds” [6]. In this article, we argue that such quantifications of the genetic contribution to disease are generally just as problematic as bad luck estimates. We stress the differences in calculation and interpretation between two seemingly similar but quite different measures, the heritability coefficient and the population attributable fraction. As motivating examples, we use studies of individual genetic variants and studies of the aggregated impact of the whole genome.

## Notation and framework

We first consider genetic variation at a specific locus, where *A* denotes the risk variant and *a* the normal (reference) variant. The risk allele frequency is *p*, implying that the genotypes *aa*, *aA* and *AA* can be expected to occur with frequency $(1 - p)^{2}$, $2p(1 - p)$, and $p^{2}$, respectively, in the population. Let *R*_0_ be the background risk for disease during a specific follow up period in the reference group with individuals without the risk variant (i.e. genotype *aa*). *RR* denotes the relative risk for disease due to carrying one copy of the risk variant versus none. A multiplicative model is assumed, implying that the relative risk due to carrying two risk variants is *RR* · *RR* = *RR*^2^. The overall risk for disease in the population, *R*_*Pop*_ can be calculated as a weighted average of the three genotype-specific risks

$$R_{Pop} = (1 - p)^{2} \cdot R_{0} + 2p(1 - p) \cdot RR \cdot R_{0} + p^{2} \cdot RR^{2} \cdot R_{0} .$$

More generally, the overall risk for disease can be averaged over *k* mutually exclusive genetic risk groups over the whole genome as

$$R_{Pop} = \sum\limits_{i = 0}^{k - 1} {p_{i} \cdot R_{i} } ,$$

where *p*_*i*_ and *R*_*i*_ represents the prevalence and risk associated with group *i* (group 0 is the reference group with background risk *R*_0_).

**Fig. 1:**
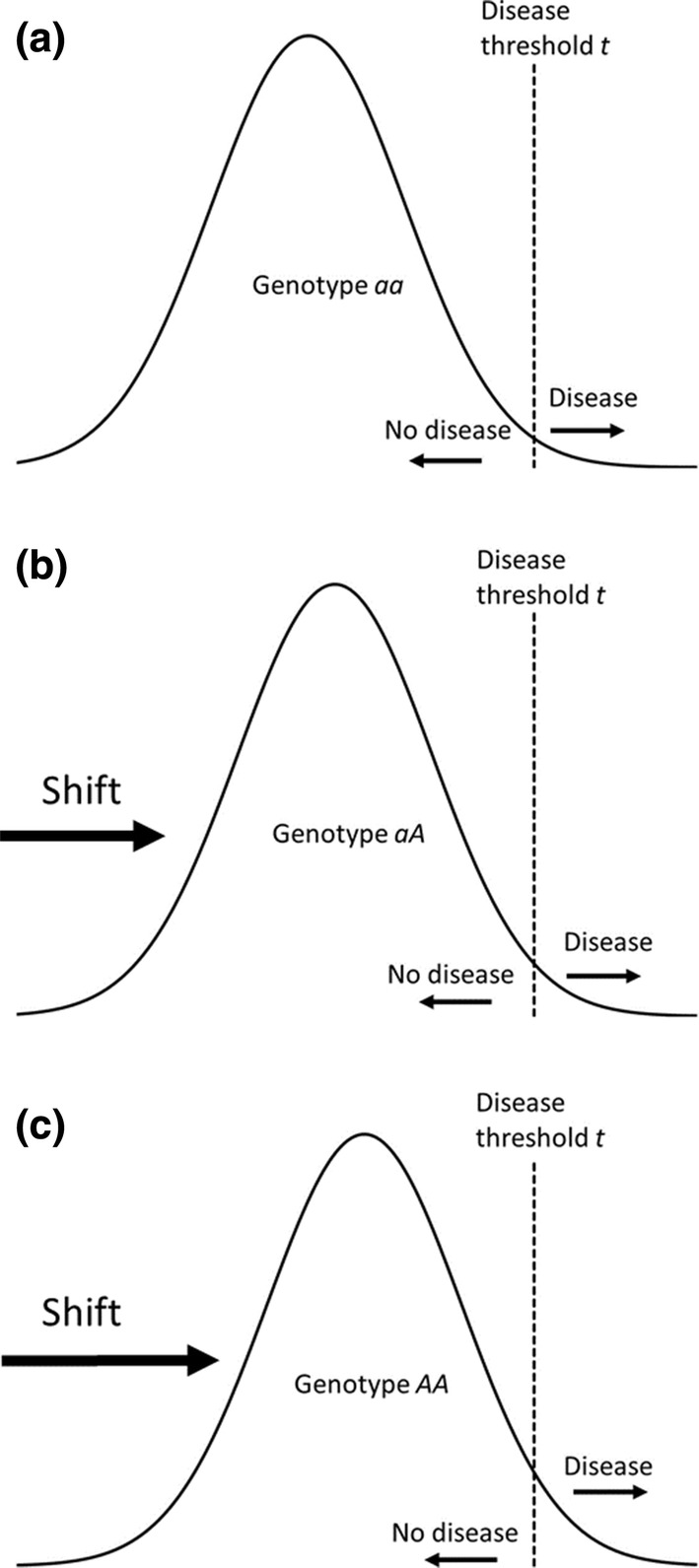
Conceptual illustration of the heritability model for a genetic variant *A* at a specific locus. The distribution of the liability is assumed to be normally distributed with the same variance in the three genotype groups. **a** Genotype *aa* (reference group) **b** Genotype *aA***c** Genotype *AA*. The shifts in liability is determined by the magnitude of the increased risk associated with *A*. Disease is assumed to occur if the liability exceed a certain threshold *t* (vertical dashed lines)

Heritability models based on analysis of variance have for long been used in genetics to study quantitative traits in the population that are subject to genetically determined differences across individuals [7]. The quantitative trait can either be observable, such as height, intelligence, lipid levels, or blood pressure, or be an underlying, unobserved trait (referred to as *liability*) that is assumed to give rise to an observed binary trait (e.g. disease or no disease) above a certain threshold. We use standard assumptions in heritability models [7], i.e. the underlying liability is normally distributed with the same variance and the same threshold for disease within all genetic risk groups. Excess disease risk due to genetic variation leads to shifts in the liability distribution curves across the groups. The size of the shift is in the case of genetic variation at a specific locus determined by the allele effect *RR* (Fig. 1).

## Heritability coefficient

The heritability coefficient *h*^2^ can for binary disease events be interpreted as the estimated proportion of the variance in disease risk that can be attributed to genetic variation. To calculate *h*^2^ we let *V*_*Pop*_ denote the overall variance in liability in the population and *V*_0_ the corresponding variance in the reference group. For simplicity we let *V*_0_ = 1 and thus *V*_*Pop*_ > 1 if genetic variation in the population increases the variance in disease risk. If the effect of the risk allele could be prevented in a way that leaves the contribution of other sources (including the environment) to the variance in disease risk unchanged (cf. Lewontin [8]), then the overall variance in the liability in the population would decrease from *V*_*Pop*_ to 1. The heritability coefficient reflects this relative contribution of genetic variation to the overall variance in liability:

$$h^{2} = \frac{{V_{Pop} - V_{0} }}{{V_{Pop} }} = \frac{{V_{Pop} - 1}}{{V_{Pop} }}.$$

## Population attributable fraction

The population attributable fraction (PAF) is the proportion of the disease events in a population that can be attributed to genetic or environmental risk factors [9–11]. PAFs can be calculated for specific risk factors (e.g. specific genetic variants or lifestyle factors such as smoking), but can also be used to assess aggregated effects of, e.g., the whole genome [12]. Using the previous notation, the overall risk for disease in the population would decrease from *R*_*Pop*_ to the background risk *R*_0_ if the excess risk due to the genetic variation could be prevented. PAF provides an estimate of the proportion of disease events that would disappear:

$$PAF = \frac{{R_{Pop} - R_{0} }}{{R_{Pop} }}.$$

## Example 1: genetic variation at a specific locus

Suppose the background risk *R*_0_ is 0.01 (1%) during a specific follow up period. A risk allele *A* that is occurring with 20% frequency increases the risk for disease two times per copy, i.e. *p* = 0.20 and *RR* = 2.0.

Thus,

$$R_{Pop} = 0.8^{2} \cdot 0.01 + 2 \cdot 0.2 \cdot 0.8 \cdot 2 \cdot 0.01 + 0.2^{2} \cdot 2^{2} \cdot 0.01 = 0.0144,$$

i.e. the overall average risk in the population is 1.44%. The genetic variation associated with *A* only leads to a marginal increase in the overall variance in liability:

$$V_{Pop} \approx 1.0249\;\left( {{\text{see}}\;{\text{Supplementary}}\;{\text{file}}\;{\text{for}}\;{\text{computational}}\;{\text{details}}} \right).$$

**Fig. 2:**
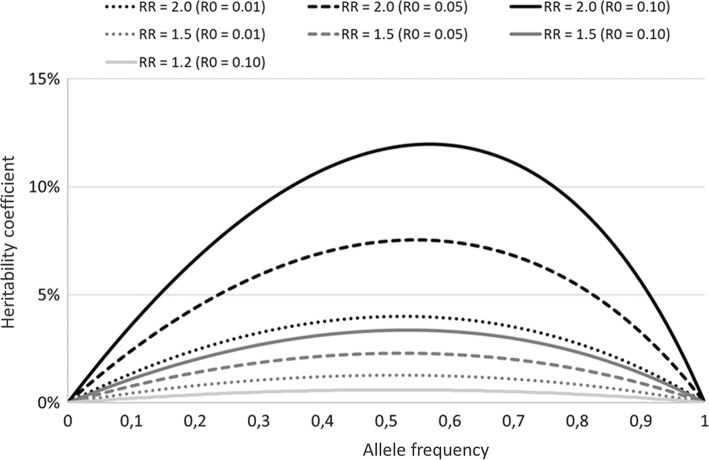
The association between risk allele frequency (*p*) and heritability coefficient (*h*^2^) at various levels of the relative risk (*RR*; 1.2, 1.5 or 2.0) for disease per copy of the risk allele and the baseline risk (*R*_0_; 0.01, 0.05 or 0.10). For *RR* = 1.2 only *R*_0_ = 0.10 is shown since *h*^2^ is marginal when *R*_0_ < 0.10 at this effect level

**Fig. 3:**
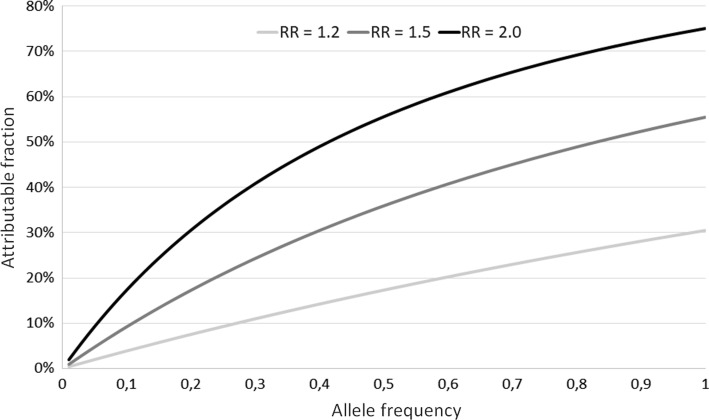
The association between risk allele frequency (*p*) and attributable fraction (AF) at various levels of the relative risk (*RR*; 1.2, 1.5 or 2.0) for disease per copy of the risk allele

The associated heritability coefficient is

$$h^{2} = \frac{{V_{Pop} - 1}}{{V_{Pop} }} \approx \frac{1.0249 - 1}{1.0249} \approx 0.024 = 2.4\% ,$$

i.e. 2.4% of the total population variance in disease risk (liability) is attributable to the risk allele. PAF reflects the relative reduction in disease risk that would occur if the effect of *A* could be prevented:

$$PAF = \frac{{R_{Pop} - R_{0} }}{{R_{Pop} }} = \frac{0.0144 - 0.01}{0.0144} \approx 0.31 = 31\% ,$$

i.e. 31% of the cases occurring in the population has the risk allele *A* as a component cause [13], and would therefore not occur if an intervention could successfully prevent its excess risk. The *h*^2^ increases to 4.4% if *R*_0_ = 0.05 and to 6.6% if *R*_0_ = 0.10. The dependence of the heritability coefficient *h*^2^ on the background risk *R*_0_, in addition to the risk allele frequency *p* and *RR*, is illustrated in Fig. 2. Maximum in *h*^2^ for a given combination of *R*_0_ and *RR* is reached for values of *p* between 50 and 60%. The magnitude of the PAF increases monotonically based on *p* and *RR*, but is independent of *R*_0_ (Fig. 3).

## Example 2: genetic variation over the whole genome

**Fig. 4:**
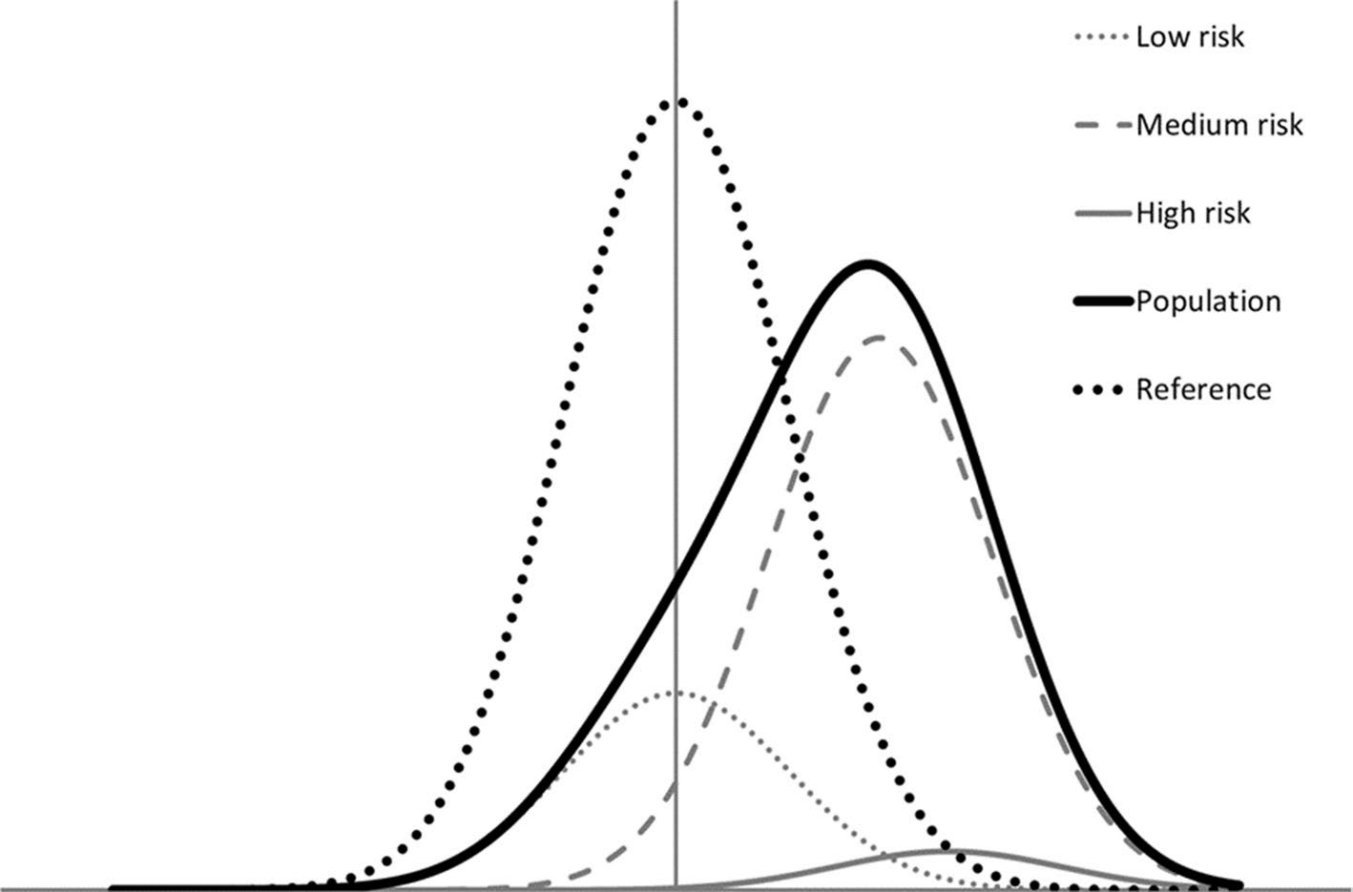
Impact of genetic variation on the liability distribution curves in example 2 (see text) with three genetic risk groups: low (risk 0.1%, prevalence 25%; dotted grey), medium (risk 10%, prevalence 70%; dashed grey) and high risk (risk 25%, prevalence 5%; solid grey curve). The solid black curve represents the liability in the overall population and the dotted black curve the reference distribution, i.e. the corresponding liability distribution in a population without this genetic variation

Next we consider genetic variation over the whole genome. To simplify, we assume that the population can be divided into three risk groups: low (*R*_0_ = 0.1%, prevalence *p*_0_ = 25%), medium (*R*_1_ = 10%, *p*_1_ = 70%) and high risk (*R*_2_ = 25%, *p*_2_ = 5%). The resulting liability distribution curves are illustrated in Fig. 4, where the location of each curve is determined by the risk and the mode (height) is determined by the prevalence. The average population risk is

$$R_{Pop} = 0.25 \cdot 0.001 + 0.70 \cdot 0.1 + 0.05 \cdot 0.25 = 0.08275 \approx 8.3\% ,$$

and the overall variance in liability is

$$V_{Pop} \approx 1.6583\;\left( {{\text{see}}\;{\text{Supplementary}}\;{\text{file}}\;{\text{for}}\;{\text{computational}}\;{\text{details}}} \right).$$

Thus, the associated heritability coefficient is

$$h^{2} = \frac{{V_{Pop} - 1}}{{V_{Pop} }} \approx \frac{1.6583 - 1}{1.6583} \approx 0.40 = 40\% ,$$

i.e. 40% of the total population variance in disease risk (Fig. 4; solid black curve) is attributable to the genetic variation. The corresponding PAF can either be calculated by assuming that only the excess risk in the group with high risk can be prevented (average population risk decreases from 8.3 to 7.03%)

$$PAF = \frac{0.08275 - 0.0703}{0.08275} \approx 0.15 = 15\% ,$$

or by assuming that all excess genetic risk (both in the medium and in the high risk group can be prevented (average population risk decreases from 8.3% down to the background risk 0.1%)

$$PAF = \frac{0.08275 - 0.001}{0.08275} \approx 0.99 = 99\% .$$

Thus, inhibiting the excess high risk would prevent 15% of all cases whereas inhibition of both excess high and medium risk would prevent 99% of all cases.

## Why these two measures may differ so much

It may seem counterintuitive that a risk allele that only contributes to 2.4% of the variance in disease risk (*h*^2^) as in example 1 still can be a component cause in 31% of all disease events (PAF). The explanation is that numerically small shifts in liability may be relatively unimportant for the increase in variance but have a large impact on the number of individuals that exceed the disease threshold in the tail of the liability distribution and become cases. In example 2, genetic variation contributed substantially (40%) to the variance in liability. The corresponding PAF ranged between 15 and 99% depending on how much of the excess genetic risk that could be prevented. Hence *h*^2^ and PAF capture different aspects of the genetic contribution, i.e. the effect on variability in risk (*h*^2^) versus the effect on average risk (PAF) [14]. In other words, a low *h*^2^ as in example 1 suggests that the risk allele contributes little to the variance in population risk. On the other hand, a high PAF implies that a substantial reduction in the average population risk would occur if a successful intervention could wipe out the excess risk [15].

## Discussion

Variance is a statistical measure that is hard to interpret even for observed traits, and not only because its unit is the square of the original measurement unit (e.g. for blood pressure the variance is expressed in (mmHg)^2^). Estimating the variance from unobservable constructs, such as liability in the heritability coefficient *h*^2^ for binary disease traits, adds further complexity to the interpretation. Calculation of *h*^2^ and similar measures of explained variance sometimes yield seemingly paradoxical results. One such example is Crohn’s disease where the risk variant at rs11209026 is very common in the population (prevalence above 90%) and most individuals thus have the same elevation in risk. This implies that *h*^2^ for this allele is close to zero as it contributes little to the population variance in risk, even though the relative risk for disease due to carrying one copy of the risk variant versus none exceeds two [16]. Similarly, smoking would explain zero percent in the variance in lung cancer risk in a population where everyone smokes equally much, but would nevertheless be the major cause of lung cancer in that population. Measures based on analysis of variance may in many situations lead to flawed take home messages (e.g. “Cancer is a matter of bad genes or bad luck—life style makes little or no difference”) [3]. For these reasons, we advise against the use of *h*^2^ and similar measures that originate from analysis of variance when communicating results to the media and the public.

Measures based on analysis of variance were classified as useless for genetic research by Lewontin already in 1974 [8], but are still commonly used also in epidemiology. If interpreted correctly, we believe that *h*^2^ can have some value as a complementary measure to PAF in scientific discussions, as these two measures describe different aspects of the genetic and environmental contribution to disease risk [14]. It is however important to stress that the purpose of the calculations are different. PAF is a useful measure in an analysis of causes and provides estimates of public health effects of interventions. By contrast, analysis of variance cannot be used to analyse causes or to estimate meaningful public health effects, as its result is determined in an intricate way by the present distribution of both environmental exposures and genotypes in the population [8].

Concerns have been raised against PAF as a measure to assess the genetic component in disease. One argument put forward is that PAF generally leads to higher numerical estimates of the genetic contribution, not only compared to *h*^2^ but often also higher than other genetic measures such as the sibling recurrence risk explained, the proportion of genetic variance explained on a log relative risk scale and the proportion of the area under the receiver-operating curve (AUC) explained [16]. PAF-values of 80% or above have even been referred to as “astonishing” [16]. However, the relationship between *h*^2^ and PAF is complex and depends on several parameters including the background risk for disease [15]. Under certain circumstances it has been shown that *h*^2^ can be higher than PAF [15]. The numerical differences can be better understood if we phrase explicitly the questions being asked by each measure (1) PAF: *What estimated fraction of all cases have the genetic risk factor as a component cause?*, (2) *h*^2^: *What estimated fraction of all variance in liability (disease risk) can be attributed to the genetic risk factor?* Thus, what we mean by genetic “contribution” to disease clearly depends on the type of ruler we are using [16].

In studies of multiple exposures (e.g. multiple genetic variants), it may seem disturbing that PAFs calculated for each exposure separately can exceed 100% if they are summed up. However, this should only be disturbing if we are studying causes that are mutually exclusive [8]. In all situations with multiple causes of disease there is no contradiction between statements such as “factor A is a cause in 50% of the cases” and “factor B in 75% of the cases” [13]. Thus, one cannot generally partition causes into fractions (such as genetic, environmental and random) that add up to 1.0 [2]. Similarly there is not conflict per se in claiming that (1) smoking causes a strong increase in lung cancer risk and (2) who develops lung cancer among the smokers is to a large extent (at least with present knowledge) a random process [3]. In order to estimate the combined contribution of different exposures (e.g. multiple genetic variants) to the disease load we have to model, or more preferably observe, their joint effects on disease [17].

In conclusion, we advise against the use of all measures based on analysis of variance when communicating the impact of a specific factor on disease risk, as they are often misunderstood in relation to what can make a difference for individual disease risk. The fraction of all disease events that could potentially be prevented is a measure that is both more relevant from a public health perspective and easier to understand.

## Electronic supplementary material

Below is the link to the electronic supplementary material.

Supplementary material 1 (DOCX 79 kb)
