# Supplementary material for: Commonly used estimates of the genetic contribution to disease are subject to the same fallacies as bad luck estimates
Source: Eur J Epidemiol. 2019 Oct 22;34(11):987–92. doi: 10.1007/s10654-019-00573-8 (PMC6861200; doi:10.1007/s10654-019-00573-8)
Supplement: Supplementary file 1 — Supplementary material 1 (DOCX 79 kb) [file 10654_2019_573_MOESM1_ESM.docx]

### Supplementary file – Calculation of heritability

We consider a population with *k* mutually exclusive genetic risk groups where the underlying liability is normally distributed with the same variance and the same threshold for disease within the groups. In the reference group (group 0), the liability is standard normal distributed N(mean *$\mu_{0}=0$*, variance *$V_{0}=1$*). Disease occurs if *L* exceed the threshold *t*, defined such that
 *$t=\Phi(1-R_{0})$*

where *R_0_* is the background risk for disease during a specific follow up period and** is the inverse of the standard normal cumulative distribution. In group *i* (*i* > 0) with risk *R_i_* there are shifts in the mean liability *L* from *µ_0_* = 0 to *µ_i_*, such that
 *$\mu_{i}=\Phi(1-R_{0})-\Phi(1-R_{i})$.*^11^

The overall mean liability *µ* is a weighted average of the group-specific means

*$\mu=\sum_{i=0}^{k} p_{i}\cdot\mu_{i}$*

and the overall variance in liability *V_Pop_* is

*$V_{Pop}=1+\sum_{i=0}^{k-1} p_{i}\cdot(\mu_{i}-\mu)^{2}$*.

### Example 1 – Genetic variation at a specific locus

With risk allele frequency *p* = 0.20 and *RR* = 2.0 and with background risk *R_0_* = 0.01, we get

*$t=\Phi(1-0.01)\approx2.326$*

*$p_{0}=(1-0.2)^{2}=0.64$*, *$p_{1}=2\cdot0.2\cdot0.8=0.32$*, *$p_{2}=0.2^{2}=0.04$*

*$\mu_{1}=\Phi(1-0.01)-\Phi(1-0.01\cdot2.0)\approx2.326-2.054\approx0.27$*

*$\mu_{2}=\Phi(1-0.01)-\Phi(1-0.01\cdot2.0^{2})\approx2.326-1.751\approx0.58$*

*$\mu=0.64\cdot0+0.32\cdot\mu_{1}+0.04\cdot\mu_{2}\approx0.11$*

*$V_{Pop}=1+0.64\cdot\mu^{2}+0.32\cdot(\mu_{1}-\mu)^{2}+0.04\cdot(\mu_{2}-\mu)^{2}\approx1.0249$*.

Thus,

$h^{2}=\frac{V_{Pop}-1}{V_{Pop}}\approx\frac{1.0249-1}{1.0249}\approx0.024=2.4\%$.

### Example 2 –Genetic variation over the whole genome

The population is divided into three genetic risk groups: low (*R_0_* = 0.1% , prevalence *p_0_* = 25%), medium (*R_1_* = 10% , *p_1_* = 70%) and high risk (*R_2_* = 25% , *p_2_* = 5%).

*$t=\Phi(1-0.001)\approx3.090$*

*$\mu_{1}=\Phi(1-0.001)-\Phi(1-0.1)\approx3.090-1.282\approx1.81$*

*$\mu_{2}=\Phi(1-0.001)-\Phi(1-0.25)\approx3.090-0.674\approx2.42$*

*$\mu=0.25\cdot0+0.70\cdot\mu_{1}+0.05\cdot\mu_{2}\approx1.39$*

*$V_{Pop}=1+0.25\cdot\mu^{2}+0.70\cdot(\mu_{1}-\mu)^{2}+0.05\cdot(\mu_{2}-\mu)^{2}\approx1.6583$*.

Thus,

$h^{2}=\frac{V_{Pop}-1}{V_{Pop}}\approx\frac{1.6583-1}{1.6583}\approx0.40=40\%$.
